# Differential Effects of Military Training on Fat-Free Mass and Plasma Amino Acid Adaptations in Men and Women

**DOI:** 10.3390/nu4122035

**Published:** 2012-12-18

**Authors:** Lee M. Margolis, Stefan M. Pasiakos, J. Philip Karl, Jennifer C. Rood, Sonya J. Cable, Kelly W. Williams, Andrew J. Young, James P. McClung

**Affiliations:** 1 Military Nutrition Division, United States Army Research Institute of Environmental Medicine, 15 Kansas Street, Natick, MA 01760, USA; E-Mails: lee.margolis@us.army.mil (L.M.M.); stefan.pasiakos@us.army.mil (S.M.P.); james.p.karl@us.army.mil (J.P.K.); andrew.j.young@us.army.mil (A.J.Y.); 2 Pennington Biomedical Research Center, Louisiana State University System, Baton Rouge, LA 70808, USA; E-Mail: jennifer.rood@pbrc.edu; 3 Experimentation and Analysis Element, Directorate of Basic Combat Training, Fort Jackson, SC 29207, USA; E-Mails: sonya.cable@us.army.mil (S.J.C.); kelly.wells.williams@gmail.com (K.W.W.)

**Keywords:** skeletal muscle, essential amino acids, branched-chain amino acids, exercise training, military training, adaptation

## Abstract

Fat-free mass (FFM) adaptations to physical training may differ between sexes based on disparities in fitness level, dietary intake, and levels of plasma amino acids (AA). This investigation aimed to determine FFM and plasma AA responses to military training, examine whether adaptations differ between male and female recruits, and explore potential associations between FFM and AA responses to training. Body composition and plasma AA levels were assessed in US Army recruits (*n* = 209, 118 males, 91 females) before (baseline) and every three weeks during basic combat training (BCT), a 10-week military training course. Body weight decreased in men but remained stable in women during BCT (sex-by-time interaction, *P* < 0.05). Fifty-eight percent of recruits gained FFM during BCT, with more (*P* < 0.05) females (88%) gaining FFM than males (36%). Total plasma AA increased (*P* < 0.05) during BCT, with greater (*P* < 0.05) increases observed in females (17%) then in males (4%). Essential amino acids (EAA) and branched-chain amino acids (BCAA) were increased (*P* < 0.05) in females but did not change in males (sex-by-time interaction, *P* < 0.05). Independent of sex, changes in EAA (*r* = 0.34) and BCAA (*r* = 0.27) from baseline were associated with changes in FFM (*P* < 0.05); greater (*P* < 0.05) increases in AA concentrations were observed for those who gained FFM. Increases in FFM and plasma AA suggest that BCT elicits a more pronounced anabolic response in women compared to men, which may reflect sex-specific differences in the relative intensity of the combined training and physiological stimulus associated with BCT.

## 1. Introduction

Skeletal muscle anabolic adaptations to physical training may be influenced by habitual dietary intake, initial fitness level, and potentially, plasma amino acid (AA) concentrations. Consuming adequate energy coupled with appropriate levels of dietary protein is requisite for long-term maintenance or gains in muscle mass, as dietary energy and protein deficiencies are associated with decrements in plasma AA levels and fat-free mass (FFM) [1,2,3]. Initial fitness level may also dictate the anabolic response to training, and although trained adults typically exhibit greater FFM and AA levels as compared to untrained adults [4], they require a greater anabolic stimulus to elicit further hypertrophic gains [5]. Plasma AA responses to exercise may also contribute to long-term anabolic adaptations to physical training. Evidence also suggests that AA responses to acute exercise may be differentially influenced by sex [6,7]. However, in contrast to acute exercise bouts, AA responses to training are not well described, and whether long-term AA responses to training and concomitant skeletal muscle adaptations differ between men and women has not been determined. 

In this study we characterize plasma AA and body composition adaptations during US Army basic combat training (BCT), which is an integrated training environment that promotes healthy dietary intake and combines daily aerobic- and resistance-type physical activities with military-specific, physically demanding tasks. Military training courses have been utilized by our group and others to characterize the relationship between nutritional status and adaptations to physical training [8,9,10]. Women initiating BCT generally exhibit lower relative fitness compared to their male counterparts [11], but experience significant gains in FFM, suggesting that the 10-week military training course elicits an anabolic response in women [9,12]. Surprisingly, few studies have reported body composition adaptations to military training in men [13], and to the best of our knowledge, no studies have reported FFM responses to BCT in a large population of US Army male recruits until a recent report from our laboratory [10]. However, sex-specific FFM comparisons in response to BCT were not described, and whether changes in plasma AA during military training were differentially influenced by sex, and contributed to changes in FFM has not been elucidated. As such, the objective of this longitudinal study was to examine whether anabolic adaptations in response to military training differ between male and female recruits, and to observe whether an association exists between plasma AA levels and FFM during BCT. We hypothesized that BCT would elicit an increase in plasma AA levels, which would be associated with changes in body composition, and that adaptation would be differentially affected by sex. 

## 2. Methods

### 2.1. Subjects

This study was approved by the Human Use Review Committee at the US Army Research Institute of Environmental Medicine, and was conducted at Fort Jackson, SC. Human volunteers participated in the study after providing informed voluntary consent. 

Test participants were US Army recruits who entered BCT in January 2010 and volunteered to participate in this longitudinal trial. The data presented in this report were collected as a part of a larger study assessing cardiometabolic risk in military recruits [10]. Anthropometrics and plasma AA concentrations were measured at baseline (week 0) and again on weeks 3, 6, and 9 to assess adaptations to each phase of BCT. A validated, semi-quantitative food frequency questionnaire (FFQ) was administered at week 0 and week 9 (Block 2005 FFQ; Nutrition Quest, Berkeley, CA) [14,15]. The FFQ was used to assess habitual energy and macronutrient intake before entering BCT (week 0) and changes in energy and macronutrient intake during training (week 9). 

### 2.2. Basic Combat Training

US Army BCT is a 10-week course comprised of physical and military training [16,17]. Physical training focuses on the development of muscular strength and aerobic capabilities through activities such as push-ups, sit-ups, weighted marches, distance running, and obstacle courses. Military training is more task specific and includes hand-to-hand combat, weapons familiarization training, and didactic course work. Male and female recruits complete the same absolute level of physical and military training. Dietary intake during BCT is ad libitum, but limited to food and beverages offered in military dining facilities, or standard military combat rations provided during field training exercises. All meals and rations were prepared and designed to comply with Military Dietary Reference Intake guidelines [18,19]. 

### 2.3. Anthropometrics

Anthropometric assessments in the study population have been previously reported and the data presented in this manuscript are used to explore relationships between body composition and plasma AA responses to BCT [10]. Briefly, body mass index (BMI) was calculated from measured height (Creative Health Products, Plymouth MI) and body mass (A&A Scales, Prospect Park, NJ). Skin fold thickness was measured at weeks 0 and 9 at the chest, triceps, and subscapular for men, and triceps, suprailiac, and abdomen for women using Lange calipers (Beta Technology, Santa Cruz, CA). Body composition was estimated using the 3-site skinfold Jackson-Pollock equation [20,21]: 

Men;

1.1125025 − 0.0013125 (sum of skinfolds) + 0.0000055 (sum of skinfolds)^2^ − 0.000244 (age)

Women;

1.089733 − 0.0009929 (sum of skinfolds) + 0.0000025 (sum of skinfolds)^2^ − 0.0000979 (age)

Our group has verified the use of 3-site skinfold measurements to estimate FFM in previous military training studies [9]. All measurements were performed under similar experimental conditions by the same trained technician. 

### 2.4. Amino Acid Analysis

Blood samples were collected after an overnight fast by antecubital venipuncture into tubes containing sodium heparin (Vacutainer; Becton Dickson, Franklin Lakes, NJ). Plasma was isolated, frozen, and shipped to the Pennington Biomedical Research Center (Baton Rouge, LA) for amino acid analysis using HPLC and *o*-phthaldialdehyde post-column derivatization (Agilent 1000 Series HPLC; Agilent Technologies).

### 2.5. Statistical Analysis

Mixed model repeated measures ANOVA (sex and time) were used to examine changes in FFQ derived energy and macronutrient intake, plasma amino acids, and body composition. Akaike’s information criteria were used to determine appropriate covariance structures. Following observation of a significant sex-by-time interaction, post-hoc comparisons were completed using Bonferroni adjustments. Dichotomous variables were created to identify the percentage of recruits that gained FFM. Sex differences for dichotomous variables were assessed using Fisher’s Exact tests. Spearman’s correlation coefficients were used to examine relationships between the percent change in FFM and plasma AA levels from week 0 and week 9. All data were analyzed using SPSS version 18.0 (SPSS Inc., Chicago, IL). Descriptive statistics are presented as mean ± SD. Significance was set at *P* ≤ 0.05. 

## 3. Results

### 3.1. Subject Characteristics

A total of 118 male and 91 female recruits volunteered to participate in this study. Male and female recruits were of similar age; however, males were taller, heavier, and had a higher BMI than female recruits (Table 1; *P* < 0.05). 

### 3.2. Body Composition

Body mass and body composition were affected by BCT [10]. Women remained weight stable from week 0 to week 9, whereas men lost 3.7 ± 5.2 kg body mass during the course (sex-by-time interaction, *P* < 0.05, Table 1). As previously reported, 58% of all recruits gained FFM during BCT, and although men exhibited greater overall FFM than women (*P* < 0.05), a greater (*P* < 0.05) percentage of women (88%) gained FFM during BCT as compared to men (36%). 

**Table 1:** Alterations in body composition ^1^^,2^.

	Male		Female	
	Week 0	Week 9	Week 0	Week 9
Age (yrs)	23 ± 5	-	23 ± 6	-
Height (cm)	176.2 ± 7.2	-	163.1 ± 6.0 *	-
Body mass (kg)	84.0 ± 16.2	80.3 ± 12.4 ^†^	66.3 ± 8.3 *	66.4 ± 7.4 *
BMI (kg∙m^−2^)	27.0 ± 4.3	25.7 ± 3.2 ^†^	25.0 ± 2.9 *	25.1 ± 2.4
Skinfold thickness (mm)	48.9 ± 22.4	39.2 ± 17.5 ^†^	51.7 ± 23.0	44.6 ± 17.0 ^†^
Body fat (%)	14.3 ± 4.8	12.3 ± 3.5 ^†^	26.6 ± 5.6 *	22.8 ± 5.1 *^,^^†^
Fat-mass (kg)	12.3 ± 5.7	10.0 ± 3.9 ^†^	18.1 ± 5.6 *	15.4 ± 4.7 *^,^^†^
Fat-free mass (kg)	71.7 ± 11.4	70.3 ± 9.4 ^†^	48.2 ± 4.8 *	51.0 ± 5.3 *^,^^†^

^1^ Values are mean ± SD, *n* = 209 (118 males, 91 females); ^†^ Different from Week 0, *P* < 0.05; * Different from males, *P* < 0.05; ^2^ Data previously reported (Pasiakos *et al.* [10]).

### 3.3. Diet

Average energy, protein (PRO), carbohydrate (CHO) and fat (FAT) intake for all recruits before BCT was 1908 ± 956 kcal∙day^−1^, 73 ± 37 g∙day^−1^ , 231 ± 124 g∙day^−1^, and 75 ± 41 g∙day^−1^, respectively, with no differences between sex. At the conclusion of BCT, men reported consuming more (*P* < 0.05) energy, PRO, CHO, and FAT compared to week 0, while patterns of dietary intake did not change in women (Table 2). Overall, absolute energy and macronutrient intakes were greater (*P* < 0.05) in men on week 9 compared to women, yet when expressed relative to body mass, there were no sex differences. 

**Table 2:** Patterns of dietary intake before and during basic combat training based on FFQ ^1^.

	Male		Female	
	Week 0	Week 9	Week 0	Week 9
**Absolute (per day)**				
Energy (kcal)	1975 ± 909	2216 ± 777 ^†^	1824 ± 1014	1789 ± 613 *
PRO (g)	78 ± 36	87 ± 33 ^†^	69 ± 38	68 ± 23 *
CHO (g)	240 ± 124	286 ± 101 ^†^	222 ± 125	240 ± 84 *
FAT (g)	77 ± 37	85 ± 35 ^†^	73 ± 46	66 ± 26 *
**Relative (per kg body mass)**				
Energy (kcal)	24.5 ± 13.3	28.1 ± 10.7 ^†^	28.5 ± 17.9	27.2 ± 10.1
PRO (g)	1.0 ± 0.5	1.1 ± 0.4 ^†^	1.1 ± 0.7	1.0 ± 0.4
CHO (g)	3.0 ± 2.0	3.6 ± 1.4 ^†^	3.5 ± 2.2	3.6 ± 1.4
FAT (g)	1.0 ± 0.5	1.1 ± 0.5 ^†^	1.1 ± 0.8	1.0 ± 0.4

^1^ Values are mean ± SD, *n* = 152 (85 males, 67 females); ^†^ Different from Week 0, *P* < 0.05; * Different from males, *P* < 0.05.

### 3.4. Plasma Amino Acids

A main effect of time (*P* < 0.05) was observed for plasma AA concentrations, as total amino acids (TAA), non-essential amino acids (NEAA), EAA and BCAA (leucine, LEU; isoleucine, ILE; and valine, VAL) levels increased during BCT. Plasma AA concentrations were greater in men as compared to women regardless of time; however, the increase in plasma AA levels from week 0 to week 9 was greater in women (sex-by-time interaction, *P* < 0.05, Table 3). Specifically, TAA, NEAA, and EAA increased from week 0 to week 9 in women by 431 ± 338 µmol∙L^−1^, 277 ± 283 µmol∙L^−1^, and 154 ± 139 µmol∙L^−1^, respectively, whereas TAA increased in men by 128 ± 411 µmol∙L^−1^ and NEAA by 106 ± 305 µmol∙L^−1^, with no change in EAA concentrations. Similar effects were observed for total and individual BCAA, as LEU, ILE, and VAL concentrations were greater in men than in women regardless of time; however, only women demonstrated an increase during BCT (sex-by-time interaction, *P* < 0.05, Table 2). 

**Figure 1:**
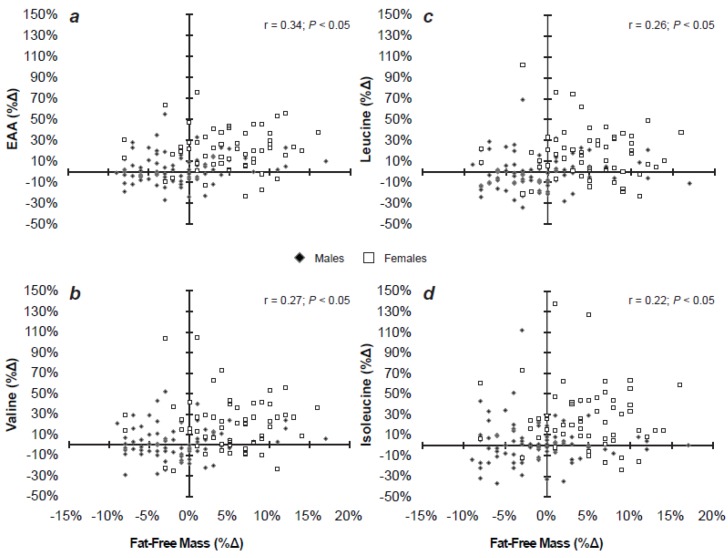
Relationships between changes in fat-free mass and plasma (**a**) essential amino acids; (**b**) valine; (**c**) leucine; (**d**) and isoleucine. *n* = 140 (81 males, 59 females).

At the initiation of BCT, FFM was associated with TAA (*r* = 0.59, *P* < 0.05), NEAA (*r* = 0.50, *P* < 0.05), EAA (*r* = 0.65, *P* < 0.05), and BCAA (*r* = 0.66, *P* < 0.05) levels for all recruits. At week 9, FFM associations with plasma EAA (*r* = 0.21, *P* < 0.05) and BCAA (*r* = 0.40, *P* < 0.05) were maintained. Furthermore, changes in FFM from weeks 0 to 9 were also associated with change in EAA (*r* = 0.34; *P* < 0.05) and BCAA (*r* = 0.27; *P* < 0.05, Figure 1a–d). The change in plasma AA levels in response to BCT was greater (*P* < 0.05) in recruits that gained FFM relative to those that did not. Specifically, EAA increased (*P* < 0.05) by 113 ± 145 µmol∙L^−1^ and BCAA by 47 ± 76 µmol∙L^−1^ in recruits who gained FFM, whereas EAA and BCAA levels did not change during BCT for those that did not gain FFM. However, correlations (FFM with AA, and change FFM with change AA) were not significant (*P* > 0.05) when men and women were assessed independently nor were TAA and NEAA levels associated with FFM at week 9. 

**Table 3:** Plasma amino acid (µmol∙L^−1^) concentrations during basic combat training in male and female recruits ^1,2,3^.

		Week 0	Week 3	Week 6	Week 9
TAA	M	3035 ± 420 ^a^	3153 ± 521 ^b^	3040 ± 360 ^a^	3162 ± 428 ^b^
	F	2522 ± 268 ^a^	2869 ± 316 ^b^	2789 ± 359 ^b^	2953 ± 318 ^c^
NEAA	M	2048 ± 297 ^a^	2179 ± 369 ^b^	2104 ± 264 ^a,b^	2155 ± 309 ^b^
	F	1761 ± 220 ^a^	2009 ± 234 ^b,c^	1947 ± 272 ^b^	2038 ± 239 ^c^
EAA	M	986 ± 153 ^a^	975 ± 187 ^a^	932 ± 124 ^b^	1008 ± 145 ^a^
	F	761 ± 90 ^a^	861 ± 119 ^b^	842 ± 118 ^b^	915 ± 114 ^b^
BCAA	M	500 ± 98 ^a^	486 ± 116 ^a^	465 ± 82 ^b^	502 ± 91 ^a^
	F	353 ± 57 ^a^	394 ± 71 ^b^	391 ± 69 ^b^	423 ± 68 ^c^
LEU	M	149 ± 26 ^a^	142 ± 33 ^a^	133 ± 21 ^b^	145 ± 22 ^a^
	F	101 ± 16 ^a^	106 ± 18 ^a,b^	107 ± 17 ^b^	117 ± 17 ^c^
ILE	M	76 ± 15 ^a^	71 ± 19 ^b^	69 ± 14 ^b^	77 ± 14 ^a^
	F	50 ± 10 ^a^	55 ± 13 ^a,b^	56 ± 11 ^a,b^	63 ± 11 ^c^
VAL	M	275 ± 61 ^a^	273 ± 72 ^a,b^	264 ± 55 ^b^	280 ± 60 ^a^
	F	202 ± 36 ^a^	233 ± 44 ^b,c^	229 ± 44 ^b^	243 ± 44 ^c^

^1^ Values are mean ± SD, *n* = 154 (87 males, 67 females); ^2^ Sex-by-time interaction, with males having higher values at all time points compared to females, *P* < 0.05; ^3^ Within-sex means not sharing the same superscripts are different (a–c), *P* < 0.05.

## 4. Discussion

The major finding from this study was that plasma AA concentrations (TAA, NEAA, EAA, and BCAA) increased over time during BCT. These data demonstrate that plasma AA and FFM responses to military training differ by sex, as the percentage of female recruits who experienced gains in FFM was greater than for males, and female recruits exhibited greater increases in plasma EAA and BCAA concentrations than male recruits. Although the mechanism accounting for these observations cannot be elucidated from this study, the data demonstrate that metabolic adaptations to the stress associated with BCT differ in male and female Soldiers.

In the current study, plasma AA levels increased over time during BCT. The response was more robust in women, particularly for those who gained FFM. The increase in plasma leucine concentrations in female recruits may be biologically significant, given that studies have demonstrated increased muscle protein anabolism in response to elevated plasma leucine levels [1,22,23]. Previous studies of healthy adults have not demonstrated increases in plasma AA in response to conventional exercise training programs. Pikosky *et al.* [24] observed reduced plasma AA concentrations (TAA, EAA, BCAA, and leucine) in response to 4-weeks of aerobic training, and Holm *et al.* [25] reported no changes in plasma AA levels following 6-weeks of aerobic-type physical training. The discrepancies between findings from previous studies and the data gleaned from the present study may largely be attributed to the differences in frequency, mode, duration, and intensity of physical training. Basic combat training is a high-volume, combined endurance and resistance-type physical training program coupled with physically strenuous military specific training, where recruits are exposed to environmental and psychological stressors. Additionally, Soldiers may travel by foot as many as 7.5 miles per day during BCT [16]. Although the level of physical activity was not quantified in the current study, the BCT course was likely more physiologically stressful than most typical physical training programs investigated in other studies. In studies of more conventional physical training program adaptations [24,25], volunteers have typically performed low to moderate intensity aerobic-type exercise bouts of 5 to 50 min, 3 to 5 times per week. Data from the current study suggest that the intensity and volume of physical training during BCT elicits an anabolic response in most Soldiers. The anabolic response is associated with elevations in plasma AA levels and concomitant increases in FFM, and this adaptation is more pronounced in female Soldiers. 

The proportion of recruits that gained FFM with associated rises in plasma AA levels is consistent with the hypothesis that BCT promotes an anabolic environment. Studies have demonstrated a linear relationship between FFM and plasma AA levels [25], which supports current findings indicating greater FFM and plasma AA levels in men than women at all time points during BCT. Interestingly, while 58% of all recruits gained FFM, more than two-thirds of those who gained FFM were women. The change in body composition for female recruits was similar to earlier reports from our laboratory documenting improved body composition in women following military training [9,12,26]. Most recently, Nindl *et al.* [9] demonstrated that female recruits maintained body weight, gained 2.8 kg of FFM, and lost 3.1 kg of body fat during BCT. Gains in FFM and reductions in body fat mass have also been reported in female Marines in response to initial military training [12]. To the best of our knowledge, no comparable reports documenting the effects of US initial military training on body composition have been conducted in men. Regardless, gains in FFM coupled with increases in plasma AA concentrations in the current study clearly suggest an anabolic effect of BCT, with women experiencing a more pronounced response than men. 

Differences in endocrine responses to exercise and pre-BCT fitness level may have contributed to the differential effects of BCT on men and women. Nindl *et al.* [9] demonstrated that BCT elicited increases in insulin-like growth factor (IGF-1) concentrations that were associated with gains in FFM in female recruits. Although IGF-1 was not measured in the present study, others have documented that endocrine responses to acute exercise or conventional physical training programs differ between men and women [27,28,29]. As such, BCT may have caused an increase in IGF-1 levels that was more prevalent in women than men, thereby leading to the more pronounced accretion of FFM. It is also possible that female Soldiers in the current study were less physically fit than males at the start of BCT. Although baseline fitness was not assessed in the present study, previous studies have demonstrated that women entering BCT having lower relative and absolute aerobic fitness and muscular strength compared to men [11]. Muscle protein turnover data in response to a fixed exercise stress suggest that greater anabolic responses to exercise training may be more pronounced in untrained *versus* trained individuals [5,30]. If women entering BCT are less physically fit than men, the cumulative physiological strain of a 10-week training program may be a more profound stimulus for anabolic adaptations in women, given that all recruits perform approximately the same level of physical activity, as suggested by the sex-related differences in AA mobilization, availability, and body composition that were observed in the present study.

Studies have demonstrated the collective impact of dietary protein intake and energy balance on plasma AA concentrations, protein turnover, and the subsequent regulation of skeletal muscle mass [1,31,32,33]. In the current study, female recruits experienced gains in plasma AA concentrations, in particular EAA and BCAA levels, and FFM, despite reporting no differences in patterns of dietary intake. Interestingly, men reported higher energy and dietary protein intakes during BCT but demonstrated no changes in plasma EAA and BCAA levels. We recognize that the use of FFQ may limit the ability to interpret dietary intake data, especially if questionnaires were not completed accurately, which has been previously reported to occur in this population [34]. To minimize the subjective aspects of the questionnaire we utilized a validated instrument and provided registered dietitians to answer questions during survey administration. To fully understand the effects of dietary protein on the observed increase in plasma AA and FFM during BCT, a more detailed method of assessing dietary intake should be utilized in future investigations. Although men increased energy intake during BCT, they experienced a reduction in body mass that women did not, which suggests that men were in a state of energy deficit. Energy deficit could have affected plasma AA and FFM responses to training in men. However, further study is required to more accurately determine how changes in habitual dietary intake may contribute to body composition adaptations to military training. 

## 5. Conclusions

This longitudinal, observational study demonstrated that FFM and plasma AA responses to BCT appear to differ between men and women, as women exhibited more pronounced changes in plasma AA levels and gains in FFM than males. The differential adaptations in women may reflect differences in energy balance, initial training status, or the intensity of the training stimulus associated with BCT. 

## References

[1] Pasiakos S.M., Vislocky L.M., Carbone J.W., Altieri N., Konopelski K., Freake H.C., Anderson J.M., Ferrando A.A., Wolfe R.R., Rodriguez N.R. (2010). Acute energy deprivation affects skeletal muscle protein synthesis and associated intracellular signaling proteins in physically active adults. J. Nutr..

[2] Weinheimer E.M., Sands L.P., Campbell W.W. (2010). A systematic review of the separate and combined effects of energy restriction and exercise on fat-free mass in middle-aged and older adults: Implications for sarcopenic obesity. Nutr. Rev..

[3] Wolfe R.R. (2002). Regulation of muscle protein by amino acids. J. Nutr..

[4] Einspahr K.J., Tharp G. (1989). Influence of endurance training on plasma amino acid concentrations in humans at rest and after intense exercise. Int. J. Sports Med..

[5] Kim P.L., Staron R.S., Phillips S.M. (2005). Fasted-state skeletal muscle protein synthesis after resistance exercise is altered with training. J. Physiol..

[6] Lamont L.S. (2005). Gender differences in amino acid use during endurance exercise. Nutr. Rev..

[7] Tarnopolsky M.A. (2000). Gender differences in substrate metabolism during endurance exercise. Can. J. Appl. Physiol..

[8] Etzion-Daniel Y., Constantini N., Finestone A.S., Shahar D.R., Israeli E., Yanovich R., Moran D.S. (2008). Nutrition consumption of female combat recruits in army basic training. Med. Sci. Sports Exerc..

[9] Nindl B.C., McClung J.P., Miller J.K., Karl J.P., Pierce J.R., Scofield D.E., Young A.J., Lieberman H.R. (2011). Bioavailable IGF-I is associated with fat-free mass gains after physical training in women. Med. Sci. Sports Exerc..

[10] Pasiakos S.M., Karl J.P., Lutz L.J., Murphy N.E., Margolis L.M., Rood J.C., Cable S.J., Williams K.W., Young A.J., McClung J.P. (2012). Cardiometabolic risk in US Army recruits and the effects of basic combat training. PLoS One.

[11] Sharp M.A., Patton J.F., Knapik J.J., Hauret K., Mello R.P., Ito M., Frykman P.N. (2002). Comparison of the physical fitness of men and women entering the U.S. Army: 1978–1998. Med. Sci. Sports Exerc..

[12] Lieberman H.R., Kellogg M.D., Bathalon G.P. (2008). Female marine recruit training: Mood, body composition, and biochemical changes. Med. Sci. Sports Exerc..

[13] Williams A.G. (2005). Effects of basic training in the British Army on regular and reserve army personnel. J. Strength. Cond. Res..

[14] Block G., Hartman A.M., Dresser C.M., Carroll M.D., Gannon J., Gardner L. (1986). A data-based approach to diet questionnaire design and testing. Am. J. Epidemiol..

[15] Block G., Woods M., Potosky A., Clifford C. (1990). Validation of a self-administered diet history questionnaire using multiple diet records. J. Clin. Epidemiol..

[16] Knapik J.J., Darakjy S., Hauret K.G., Canada S., Marin R., Jones B.H. (2007). Ambulatory physical activity during United States Army basic combat training. Int. J. Sports Med..

[17] Knapik J.J., Sharp M.A., Darakjy S., Jones S.B., Hauret K.G., Jones B.H. (2006). Temporal changes in the physical fitness of US Army recruits. Sports Med..

[18] (2001). AR 40-25. Nutrition Standards and Education.

[19] (2007). Pamphlet 30-22. Operationg Procedures for the Army Food Program.

[20] Jackson A.S., Pollock M.L. (1978). Generalized equations for predicting body density of men. Br. J. Nutr..

[21] Jackson A.S., Pollock M.L., Ward A. (1980). Generalized equations for predicting body density of women. Med. Sci. Sports Exerc..

[22] Drummond M.J., Glynn E.L., Fry C.S., Timmerman K.L., Volpi E., Rasmussen B.B. (2010). An increase in essential amino acid availability upregulates amino acid transporter expression in human skeletal muscle. Am. J. Physiol. Endocrinol. Metab..

[23] Pasiakos S.M., McClung J.P. (2011). Supplemental dietary leucine and the skeletal muscle anabolic response to essential amino acids. Nutr. Rev..

[24] Pikosky M.A., Gaine P.C., Martin W.F., Grabarz K.C., Ferrando A.A., Wolfe R.R., Rodriguez N.R. (2006). Aerobic exercise training increases skeletal muscle protein turnover in healthy adults at rest. J. Nutr..

[25] Holm G., Sullivan L., Jagenburg R., Bjorntorp P. (1978). Effects of physical training and lean body mass of plasma amino acids in man. J. Appl. Physiol..

[26] Karl J.P., Lieberman H.R., Cable S.J., Williams K.W., Young A.J., McClung J.P. (2010). Randomized, double-blind, placebo-controlled trial of an iron-fortified food product in female soldiers during military training: relations between iron status, serum hepcidin, and inflammation. Am. J. Clin. Nutr..

[27] Kraemer W.J., Staron R.S., Hagerman F.C., Hikida R.S., Fry A.C., Gordon S.E., Nindl B.C., Gothshalk L.A., Volek J.S., Marx J.O., Newton R.U., Hakkinen K. (1998). The effects of short-term resistance training on endocrine function in men and women. Eur. J. Appl. Physiol. Occup. Physiol..

[28] Tarnopolsky M.A. (2008). Sex differences in exercise metabolism and the role of 17-beta estradiol. Med. Sci. Sports Exerc..

[29] Vislocky L.M., Gaine P.C., Pikosky M.A., Martin W.F., Rodriguez N.R. (2008). Gender impacts the post-exercise substrate and endocrine response in trained runners. J. Int. Soc. Sports Nutr..

[30] Tang J.E., Perco J.G., Moore D.R., Wilkinson S.B., Phillips S.M. (2008). Resistance training alters the response of fed state mixed muscle protein synthesis in young men. Am. J. Physiol. Regul. Integr. Comp. Physiol..

[31] Calloway D.H., Spector H. (1954). Nitrogen balance as related to caloric and protein intake in active young men. Am. J. Clin. Nutr..

[32] Friedlander A.L., Braun B., Pollack M., MacDonald J.R., Fulco C.S., Muza S.R., Rock P.B., Henderson G.C., Horning M.A., Brooks G.A., Hoffman A.R., Cymerman A. (2005). Three weeks of caloric restriction alters protein metabolism in normal-weight, young men. Am. J. Physiol. Endocrinol. Metab..

[33] Pikosky M.A., Smith T.J., Grediagin A., Castaneda-Sceppa C., Byerley L., Glickman E.L., Young A.J. (2008). Increased protein maintains nitrogen balance during exercise-induced energy deficit. Med. Sci. Sports Exerc..

[34] Tanskanen M., Uusitalo A.L., Häkkinen K., Nissilä J., Santtila M., Westerterp K.R., Kyröläinen H. (2009). Aerobic fitness, energy balance, and body mass index are associated with training load assessed by activity energy expenditure. Scand. J. Med. Sci. Sports.

